# The effects of an innovative GP-physiotherapist partnership in improving COPD management in primary care

**DOI:** 10.1186/s12875-023-02097-3

**Published:** 2023-07-10

**Authors:** Lisa Pagano, Sarah Dennis, Sally Wootton, Andrew S. L. Chan, Nicholas Zwar, Sriram Mahadev, Deborah Pallavicini, Zoe McKeough

**Affiliations:** 1 Sydney School of Health Sciences, Faculty of Medicine and Health, University of Sydney, Sydney, Australia; 2 Ingham Institute for Applied Medical Research, Sydney, Australia; 3 South Western Sydney Local Health District, Liverpool, Australia; 4 Chronic Disease Community Rehabilitation Service, Northern Sydney Local Health District, Sydney, Australia; 5 Royal North Shore Hospital, St Leonards, Australia; 6 Northern Clinical School, University of Sydney, Sydney, Australia; 7 Faculty of Health Sciences and Medicine, Bond University, Gold Coast, Australia; 8 Sydney North Primary Health Network, St Leonards, Australia

**Keywords:** COPD, Primary care, Physical activity, Pulmonary rehabilitation, Allied health

## Abstract

**Background:**

Evidence suggests that management of people with Chronic Obstructive Pulmonary Disease (COPD) in primary care has been suboptimal, in particular, with low referral rates to pulmonary rehabilitation (PR). The aim of this study was to evaluate the effectiveness of a GP-physiotherapist partnership in optimising management of COPD in primary care.

**Methods:**

A pragmatic, pilot, before and after study was conducted in four general practices in Australia. A senior cardiorespiratory physiotherapist was partnered with each general practice. Adults with a history of smoking and/or COPD, aged ≥ 40 years with ≥ 2 practice visits in the previous year were recruited following spirometric confirmation of COPD. Intervention was provided by the physiotherapist at the general practice and included PR referral, physical activity and smoking cessation advice, provision of a pedometer and review of inhaler technique. Intervention occurred at baseline, one month and three months. Main outcomes included PR referral and attendance. Secondary clinical outcomes included changes in COPD Assessment Test (CAT) score, dyspnoea, health activation and pedometer step count. Process outcomes included count of initiation of smoking cessation interventions and review of inhaler technique.

**Results:**

A total of 148 participants attended a baseline appointment where pre/post bronchodilator spirometry was performed. 31 participants with airflow obstruction on post-bronchodilator spirometry (mean age 75yrs (SD 9.3), mean FEV_1_% pred = 75% (SD 18.6), 61% female) received the intervention. At three months, 78% (21/27) were referred to PR and 38% (8/21) had attended PR. No significant improvements were seen in CAT scores, dyspnoea or health activation. There was no significant change in average daily step count at three months compared to baseline (mean difference (95% CI) -266 steps (-956 to 423), *p* = 0.43). Where indicated, all participants had smoking cessation interventions initiated and inhaler technique reviewed.

**Conclusion:**

The results of this study suggest that this model was able to increase referrals to PR from primary care and was successful in implementing some aspects of COPD management, however, was insufficient to improve symptom scores and physical activity levels in people with COPD.

**Trial registration:**

ANZCTR, ACTRN12619001127190. Registered 12 August 2019 – Retrospectively registered, http://www.ANZCTR.org.au/ACTRN12619001127190.aspx.

**Supplementary Information:**

The online version contains supplementary material available at 10.1186/s12875-023-02097-3.

## Contributions to the Literature

- This study evaluates a novel physiotherapist-led intervention in primary care whereby experienced cardiorespiratory physiotherapists work in partnership with GPs to optimise COPD management.
- Our results show that this model has the potential to improve some aspects of the implementation of COPD management guidelines such as increased referrals to pulmonary rehabilitation programs.
- These findings contribute to current gaps in the literature surrounding potential models to improve COPD management which is of relevance to service providers and policy makers looking to adopt innovative models into practice.

## Background

Chronic obstructive pulmonary disease (COPD) is a leading cause of morbidity and mortality, placing a significant and growing burden on healthcare systems worldwide [1, 2] It is known that frequent COPD exacerbations are associated with worse health outcomes, higher mortality rates and faster decline in forced expiratory volume in one second (FEV_1_) [3–5], highlighting the importance of optimal management. It is possible that delaying time to first exacerbation in people with COPD could slow disease progression and result in better health outcomes. Diagnosis of COPD and subsequent management often occurs in primary care with the majority of patients with COPD managed by their general practitioner (GP). Guidelines have been produced and disseminated outlining optimal evidence-based management of COPD in primary care, including the COPD-X guidelines in Australia [6] and internationally, the Global Initiative for Obstructive Lung Disease (GOLD) guidelines [2]. Yet despite strong international recommendations, evidence suggests suboptimal implementation of some management strategies as well as low awareness of these guidelines by primary care practitioners [7–9].

Low referral rates to pulmonary rehabilitation (PR) is one example where implementation has been suboptimal [10–12]. PR is regarded as the cornerstone of non-pharmacological management in COPD comprising a structured program of supervised exercise training, education and behaviour change [13]. Guidelines recommend that PR should be offered to patients with COPD who are limited by shortness of breath on exertion and states that patients with COPD, of all Modified Medical Research Council (mMRC) grades, gain significant benefit from rehabilitation [6]. Yet it has been reported that referral rates of eligible patients with stable COPD range from 0–11% [10] and that within primary care, referral often occurs later in the disease process [14]. Physical inactivity in COPD is also a significant problem [15, 16] with reductions in physical activity (PA) commencing early in the disease trajectory [17]. Lower levels of PA have been linked to lower quality of life (QoL), faster decline in lung function and increased risk of hospitalisations and mortality [6, 18]. Consequently, PA interventions constitute an important component of COPD management with guidelines emphasising that people with COPD should be encouraged to be regularly physically active and to reduce sedentary behaviour time [6], yet data demonstrates that people with COPD continue to engage in low levels of PA [15, 17]. Strategies aimed at optimising disease management and increasing uptake of interventions such as PR are essential in reducing the burden of COPD on healthcare systems.

Different methods to improve implementation of COPD management have been trialled in primary care with varying success rates. Initiatives aimed at upskilling GPs and providing care bundles summarising key elements of COPD care have proven effective in increasing implementation rates of some aspects of COPD management such as smoking cessation interventions [19]. However, barriers to effective implementation of some management strategies have also been identified. For example, studies have suggested that GPs lack confidence in managing COPD and report low self-efficacy in discussions surrounding PR, PA and complex behaviour change [9, 12, 20]. Lack of time is also commonly cited as a barrier [7, 21] leading researchers to question if there is a role for other healthcare professionals to assist GPs in the management of patients with COPD.

Studies examining the utilisation of the wider multidisciplinary team (MDT) have yielded mixed results. For example, one Cochrane review found that integrated disease management which included studies with MDT teams for COPD led to improvements in disease-specific QoL, exercise capacity and a reduction in respiratory-related hospital admissions and hospital days per person [22]. Other studies have reported no significant differences in primary outcome measures such as health-related quality of life (HRQoL), symptom scores or smoking status when utilising healthcare professionals such as practice nurses [23, 24], pharmacists [25] or a mixed MDT [26]. Preliminary evidence suggests that experienced physiotherapists as a first point of contact in primary care for musculoskeletal complaints is safe and effective, yet there are currently no published studies looking at a physiotherapist integrated into primary care assisting in the management of people with COPD in Australia. Given that physiotherapists currently contribute to COPD management, such as through PA recommendations, the provision of PR programs, airway clearance techniques and spirometry interpretation, physiotherapists could be a successful option to work in partnership with GPs to implement interventions and increase referral rates to PR in primary care.

### Aims

The primary aim of the INTEGRATED (InNovaTivE Gp-physiotheRapist pArTnErship for copD) study is to implement and evaluate the effectiveness of a GP-physiotherapist partnership in optimising management of COPD in primary care. In particular, we wanted to determine if this model of care can increase referrals to PR from primary care and PR attendance. Secondarily we wanted to determine the effects of this model of care on improving clinical outcomes (including health status, symptom scores, patient health activation and PA levels) and process outcomes (including smoking status and uptake of smoking cessation interventions, influenza and pneumococcus immunisation status, review of inhaler technique, initiation of action plans and hospital utilisation).

## Methods

A pilot, pragmatic before and after study within the “health services” research domain was conducted in accordance with the published study protocol [27] (see Additional file 1). The protocol followed the TIDieR (Template for Intervention Description and Replication framework) Framework. This paper focuses on the effectiveness of this model of care on outcome measures at three months. Recruitment commenced in October 2018 and was completed in January 2020. The three-month follow-up was completed by May 2020. Ethics approval was obtained from Northern Sydney Local Health District Human Research Ethics committee and the trial was registered with the Australia and New Zealand Clinical Trials Registry (ACTRN12619001127190).

### Recruitment

Eligible general practices in Sydney, Australia were recruited with assistance from a primary health network. Once consent was obtained, a senior physiotherapist with at least five years cardiorespiratory experience was integrated into each practice. A research assistant or general practice staff identified potentially eligible participants from a search of the practice electronic records using searches developed from previous studies [23, 28]. To be considered eligible, participants met the following criteria; (i) were adults aged 40 years and over; (ii) had attended the practice at least twice with one visit in the preceding 12 months; and (iii) had a documented history of smoking (current or former smoker) in their medical notes or (iv) had a recorded diagnosis of COPD or were taking medications prescribed for COPD (i.e. short acting inhaled β2 agonists (SABA), short acting muscarinic antagonists (SAMA), long acting inhaled β2 agonists (LABA), long acting muscarinic antagonists (LAMA), combination of LABA/LAMA and/or inhaled corticosteroids). Exclusion criteria included terminal cancer, a cognitive impairment, home oxygen requirement, did not speak sufficient English, pregnancy or on clinical grounds by the GP or practice nurse.

Potentially eligible participants were invited to take part in the study via letter or phone call. After obtaining written informed consent, participants attended a baseline assessment with the senior cardiorespiratory physiotherapist at the general practice to identify or confirm the presence of COPD with airflow obstruction via pre/post bronchodilator spirometry. Those with a post-bronchodilator forced expiration in one second/forced vital capacity (FEV_1_/FVC) of < 0.7 [2] were assigned a diagnosis of COPD and were considered eligible for the intervention arm of the study.

### Intervention

All physiotherapists in the study completed an advanced training workshop in the management of COPD according to the COPD-X guidelines [6]. A brief intervention occurred at three timepoints and was coordinated by the physiotherapist at each site in collaboration with general practice staff.

All participants received the following intervention; (i) referral to PR if they met the requirement according to the COPD-X guidelines [6]; (ii) physical activity advice and counselling using the 5 A’s approach (Ask, Advise, Assess, Assist, Arrange follow-up) according to the Australian Physical Activity and Sedentary Behaviour Guidelines [29]; (iii) provision of a pedometer (Yamax 3D Pocket Pedometer, Pedometers Australia, Cannington, WA) and pedometer diary to monitor PA goals and guide exercise prescription at follow-up appointments with participants instructed to wear the pedometer every day during awake hours and record daily steps in the diary; (iv) individualised smoking cessation advice, support and appropriate referral to their GP if required; (v) review of inhaler technique if applicable; and (vi) provision of education booklets regarding PA guidelines, smoking cessation and general COPD management. The physiotherapist also worked in partnership with the GP and patient to develop or review a COPD-specific GP Management Plan (GPMP) and/or a COPD action plan. Participants then returned for a follow-up visit with the physiotherapist at one month to review PA levels and establish a PA goal to progress towards. A final assessment was completed at three months to review progress.

### Outcomes and measurements

A detailed description of each outcome measure is included in the published study protocol [27]. The primary outcome measures for this paper were number (%) eligible for PR, number (%) referred to PR and number (%) attended PR. Secondary outcome measures included clinical and process outcomes. Clinical outcomes were assessed at baseline and three months and included i) health status assessed by the COPD Assessment Test (CAT) [30]; ii) dyspnoea measured by the mMRC [31]; iii) patient health activation, which refers to an individual’s knowledge, skill and confidence for managing their health [32], assessed by the Patient Activation Measure (PAM) (0–100 score with progressively higher scores indicating higher health activation) [32, 33]; iv) number (%) meeting PA guidelines self-reported using the Active Australia Questionnaire (AAQ) [34]; v) readiness for change on the Physical Activity Stages of Change Questionnaire (PASOCQ) [35]; vi) daily step count measured by a pedometer; and vii) compliance to set step goals. Process outcomes were measured at either baseline and three months or only at the three-month timepoint. These included i) smoking status by self-report and uptake of a smoking cessation program in those who were smokers at baseline; ii) immunisation status for influenza and pneumococcus; iii) number (%) review of inhaler technique (where prescribed); iv) number (%) of GPMPs and/or action plans initiated; and v) hospital utilisation as assessed through self-report of exacerbations, hospital admissions and emergency department attendance.

Baseline data collection was completed by the physiotherapist at each practice. Three-month data collection was completed either in person or via telephone by the physiotherapist or a research assistant.

### Statistical analysis

Data was analysed using IBM SPSS Statistics for Windows, version 24.0. (IBM Corp., Armonk, N.Y., USA). Data is presented as mean and standard deviation (SD), median and interquartile range (IQR) or number (%) as appropriate. Summary descriptive statistics were calculated for baseline demographic data. Shapiro–Wilk tests were performed to determine normality distributions. For the primary outcomes of PR referral and attendance, descriptive statistics were performed. There are currently no reported targets for referral rates to PR in Australian general practice. The British Thoracic Society tasks PR providers with achieving at least 70% PR completion rate so for this paper, referral to PR and attendance of those referred of ≥ 70% of participants was considered to be clinically significant [36]. A change of two points on the CAT was considered the minimal important difference (MID) [37]. Participant PAM scores were categorised into ‘levels of activation’ (level 1: ≤ 47.0 points; level 2: 47.1–55.1 points; level 3: 55.2 to 67.0 points; level 4: ≥ 67.1 points) [38] to allow for comparison. Average improvements in the PAM score of 2.5 to 6.5 have been reported following interventional studies [39] and have been used as a guide to an appropriate level of improvement for this study given there are no reported MIDs for the PAM in people with COPD. For PA, daily step count was averaged over at least five wear days out of seven days. At three months, an increase of at least 600 steps per day was considered the MID [40] for significant change in PA. Compliance to set step goals was assessed at each week following the one-month follow-up appointment. Participants who achieved 80% or more of their set step goals were considered to be compliant. Change over time from baseline to three months within groups was analysed with Wilcoxon signed rank tests or two-tailed paired t-tests for ordinal and continuous variables respectively and with McNemars tests for dichotomous variables. The estimated between-group difference and the 95% confidence intervals (95% CI) are reported. All significance tests were two-sided and p values < 0.05 were considered statistically significant.

## Results

**Fig. 1:**
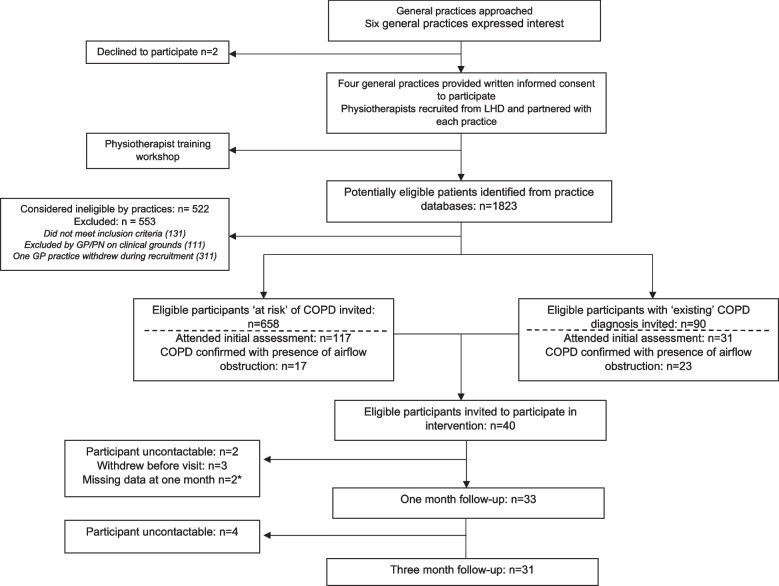
Study flow. Abbreviations: COPD: Chronic Obstructive Pulmonary Disease; GP: general practitioner; LHD: local health district. *2 participants did not attend one month assessment and returned for three-month follow-up included in analysis

Figure 1 shows the flow chart of study enrolment. In total, 148 eligible participants attended a baseline appointment with the physiotherapist where pre/post bronchodilator spirometry was performed. COPD with airflow obstruction on post-bronchodilator spirometry was detected or confirmed in 27% (40/148) of those that attended and these participants were offered the intervention. A total of 78% (31/40) of participants completed the intervention and were included in the analysis. Loss to follow-up was 23% (9/40) and there was one adverse event during the intervention period where one participant experienced a flare-up of their pre-existing knee osteoarthritis.

The baseline characteristics of participants who completed the intervention (*n* = 31) are included in Table 1. The majority of participants were classified as having mild to moderate obstruction and reported high levels of health activation according to the PAM.

**Table 1:** Baseline characteristics of subjects that completed the intervention

	**TOTAL** ***n* = 31**
**Mean age, years (SD)**	75 (9.3)
**Gender (female)**	19 (61%)
**Mean body mass index, Kg/m**^**2**^** (SD)**	27.7 (5.3)
**Born in Australia**	20 (65%)
**English spoken at home**	140 (97%)
**Identify as Aboriginal and/or Torres Strait Islander**	2 (7%)
**Currently married or de facto**	16 (52%)
**Employment status**	
Employed-full/part-time/casual	4 (13%)
Retired/pensioner	25 (81%)
Unemployed/student/disability pension/home duties/carer	2 (6%)
**Education**	
Some primary school	1 (3%)
Completed high school/some high school	12 (39%)
Tertiary education/vocational training	18 (58%)
**Smoking status**	
Current	5 (16%)
Former	24 (77%)
Never smoked	2 (7%)
**Mean number of co-morbidities (SD)**	5 (3.1)
**Mean post-bronchodilator FEV**_**1**_**/FVC (SD)**	0.61 (0.1)
**Mean post-bronchodilator FEV**_**1**_**% predicted (SD)**	75 (18.6)
**GOLD Stage I**	18 (58%)
**GOLD Stage II**	2 (6%)
**GOLD Stage III**	0 (0%)
**GOLD Stage IV**	1 (1%)

Data are presented as Number (%) unless indicated otherwise

*Abbreviations*: *COPD* Chronic Obstructive Pulmonary Disease, *FEV*_*1*_ Forced expiratory volume in one second, *FVC* Forced vital capacity, *GOLD* Global Initiative for Chronic Obstructive Lung Disease, *SD* standard deviation

COPD GOLD staging classification^2^—Stage 1: FEV_1_ ≥ 80%; Stage 2: FEV_1_ 50–79%; Stage 3: FEV_1_ 30–49%; Stage 4: FEV_1_ < 30%

### Primary outcomes

**Fig. 2:**
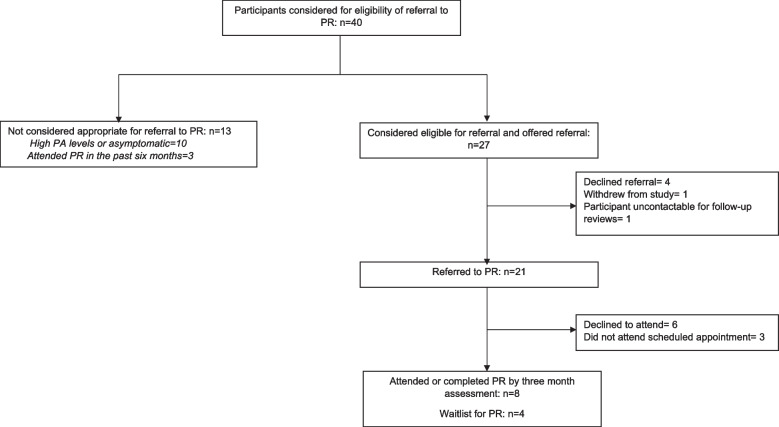
Pulmonary Rehabilitation Flow. Abbreviations: PA: physical activity; PR: Pulmonary Rehabilitation

Figure 2 details data on PR referral and attendance. Of those screened for eligibility of referral to PR, 68% (27/40) were considered eligible with reasons for ineligibility being that the participant was either asymptomatic with no exertional dyspnoea and/or had high PA levels according to the Australian Physical Activity and Sedentary Behaviour Guidelines [29]. A total of 78% (21/27) were referred to PR. PR attendance was lower than expected, with only 38% (8/21) of participants referred to PR having attended a PR program at the three-month assessment. An additional four participants were waitlisted for PR or were awaiting contact to schedule an initial appointment.

### Clinical outcomes

Results of secondary clinical outcomes are listed in Table 2. Following the intervention, there was no significant improvement in health status scores as measured by the CAT and the MID of a change in 2 points was not reached. There was also no statistically significant improvement in dyspnoea score on the mMRC at three months. Following the intervention, there was no statistically significant change in patient health activation and of those that completed the intervention, 32% increased their activation level while 52% remained the same and 16% saw a decrease in their activation level.

**Table 2:** Clinical Outcomes at baseline and three months

	**Baseline**	**Three Months**	**Mean Difference** **(95%CI)**	***P*-value**
**CAT, *n* = 29**	11.97 (7.6)	10.62 (7.6)	-1.35 (-3.3 to 0.6)	0.22
**mMRC, *n* = 30** **median [IQR]**	1 [1]	1 [1]	N/A	0.13
Number (%) score				
0	8 (28%)	10 (33%)	N/A	
1	16 (53%)	15 (50%)		
2	3 (10%)	5 (17%)		
3	3 (10%)	0 (0%)		
4	0 (0%)	0 (0%)		
**PAM, *n* = 30**	69.2 (18.1)	68.1 (17.8)	-1.12 (-5.6 to 7.8)	0.73
Number (%) level				
Level 1	3 (10%)	3 (10%)		
Level 2	4 (13%)	4 (13%)		
Level 3	10 (33%)	13 (43%)		
Level 4	13 (43%)	10 (33%)		
**AAQ time spent in activity, minutes**				
Walking *n* = 29	520 (1146)	451 (637)	-68 (-619 to 482)	0.80
Gardening *n* = 28	60 (104)	68 (92)	8 (-40 to 56)	0.74
Vigorous PA *n* = 30	117 (165)	93 (126)	-25 (-73 to 23)	0.30
Moderate PA *n* = 28	79 (162)	118 (145)	39 (-13 to 90)	0.14
**AAQ median number of sessions [IQR]**				
Walking *n* = 28	6 [6]	7 [7]	N/A	0.86
Gardening *n* = 27	0 [2]	0 [2]		0.16
Vigorous PA *n* = 29	1 [4]	2 [4]		0.95
Moderate PA *n* = 28	0 [2]	0 [3]		0.08
**AAQ meeting PA guidelines**^**a**^**, *n* = 26**				
Number (%) completing sufficient physical activity minutes per week	23 (89%)	21 (81%)	N/A	0.63
Number (%) sedentary	0 (0%)	0 (0%)		1.00
**Pedometer daily step count, *n* = 23**	6180 (3785)	5916 (3203)	-266 (-956 to 423)	0.43
**PASOCQ, *n* = 28**				
Number (%) level	2 (7%)	2 (7%)		
Pre-contemplation	5 (18%)	3 (11%)	N/A	N/A
Contemplation	6 (21%)	3 (11%)		
Preparation	1 (4%)	2 (7%)		
Decision/Action Maintenance	14 (50%)	18 (64%)		

Data presented as mean (SD) unless indicated otherwise

*Abbreviations*: *AAQ* Active Australia Questionnaire, *CAT* COPD Assessment Test, *IQR* interquartile range, *mMRC* Modified Medical Research Council Dyspnoea Scale, *PA* physical activity, *PAM* Patient Activation Measure, *PASOCQ* Physical Activity Stages of Change Questionnaire, *SD* Standard deviation

^a^Australian Physical Activity and Sedentary Behaviour Guidelines^29^

Similar proportions of participants reported engaging in sufficient PA according to the Australian Physical Activity and Sedentary Behaviour Guidelines [29] on the AAQ at baseline and three months and these differences were not significant. At three months, there was also no significant difference observed in average daily step count. Immediately following the one-month follow-up appointment, 90% of participants were compliant with set step goals however, there was a gradual decrease over time to 50% of participants being compliant with set step goals at three months.

### Process outcomes:

Table 3 shows results of secondary process outcomes. All those who were current smokers at baseline had a smoking cessation intervention initiated by the physiotherapist. At three months, the proportion of current smokers reduced compared to baseline however, this was not significant. In addition, there was no change in the number of influenza vaccinations at three months compared to baseline however, there was a trend towards an increase in number of pneumococcus vaccinations. All participants taking prescribed inhalers for medical management had their technique reviewed by the physiotherapist during the study period and 71% (22/31) reported that a GPMP and/or action plan had been developed or reviewed for them during the study period. An additional five participants reported the physiotherapist had initiated an action plan that had not yet been reviewed by the GP at the time of the three-month assessment. There were no significant differences observed in self-report of exacerbations, hospital admissions and emergency department attendance.

**Table 3:** Process Outcomes at baseline and three months

	**Baseline**	**Three Months**	***P*-value**
**Current smokers**	5 (16%)	1 (3%)	0.13
**Vaccinated for influenza**	27 (87%)	28 (90%)	1.00
**Vaccinated for pneumococcus**	18 (58%)	23 (74%)	0.06
**Inhaler technique reviewed**	Not assessed	26/26^a^(100%)	N/A
**GPMP and/or action plan initiated**	4^b^(13%)	22 (71%)	N/A
**Reported exacerbations**	0 (0%)	1 (3%)	1.00
**ED admissions for:**			
COPD	0 (0%)	1 (3%)	N/A
Heart disease	0 (0%)	1 (3%)	
Other	3 (10%)	4 (13%)	

Data presented as Number (%)

*Abbreviations*: *COPD* chronic obstructive pulmonary disease, *ED* emergency department, *GPMP* general practitioner management plan, *N/A* not applicable

^a^*n* = 26 had prescribed inhalers for COPD management

^b^4/31 participants with previous doctor diagnosis of COPD with self-reported GPMP and/or action plan

## Discussion

This study evaluated the effects of a GP-physiotherapist partnership in optimising management of COPD in primary care. Results demonstrate that a physiotherapist integrated into the primary care team was successful at improving referrals to PR and implementing some key components of COPD management, according to the COPD-X guidelines [6], such as initiating smoking cessation interventions and action plans. This, however, did not translate into improvements in patient-reported symptom scores, health activation or PA levels, with no significant improvement observed at three months compared to baseline in these outcomes.

A primary aim of this study was to increase referrals to PR from primary care. Following the intervention, close to 80% of eligible participants were referred to PR which is significantly higher than that suggested by current standards [11, 14]. This finding is important as PR has been shown to result in significant improvements in exercise capacity and HRQoL in people with COPD [41, 42]. The higher referral rate observed could be due to the fact that the physiotherapists recruited in this study had extensive experience in PR programs as well as knowledge of the referral processes. These are important points to consider as barriers to PR referral include low knowledge and awareness of PR and the benefits of PR, and low knowledge of the referral process [10, 12]. In addition, one of the most common enablers to referral and attendance is training and experience in PR [10, 43]. Therefore, the potential added value of this model is that by using a physiotherapist in primary care who is confident with discussions around PR, provides the opportunity to routinely prompt and educate on the program which is likely to increase referral and attendance to PR.

In our study, whilst PR attendance remained suboptimal at 38% by the three-month review, this again is higher than current data suggests where it is estimated that only 5–10% of Australians with moderate to severe COPD have accessed a PR program [44, 45]. Similar to current literature, our results also suggest that there are additional barriers that must be addressed beyond the clinical GP setting in order to increase PR attendance. Individual factors related to beliefs about disease and exercise as well as individual circumstances and the context of one’s own environment could have impacted on attendance [46]. For example, qualitative data has reported that patients who felt unable to cope with their condition and needed additional support were more likely to accept referral to PR [14] whereas the perception that their lung disease was not severe enough was a barrier to uptake [46]. The majority of our patient population reported low levels of activity limitation and high levels of health activation, so it is likely that they may not have felt the need to participate in PR.

A core consideration of implementation into clinical practice is whether this model of care can improve delivery of multiple key management components, thereby resulting in long-term benefit for people with COPD. The effects of smoking cessation on COPD progression are well known [47, 48] and it has been shown that initiation of self-management plans, including action plans for exacerbations, can improve HRQoL and reduce risks of respiratory-related hospitalisation in people with COPD [49]. It follows that if completion of these key aspects of care can be implemented effectively with good patient adherence, this may lead to better outcomes for patients in the long-term. In our study, the increased rate of pneumococcal vaccination, initiation or review of action plans and smoking cessation interventions suggests improved delivery of care. All participants with prescribed inhalers had their technique reviewed by the physiotherapist which is higher than data from a primary care audit conducted in Wales, that reported approximately 44% of people with COPD had evidence of an inhaler check in the previous year [50]. Our findings are encouraging and may reflect the benefit of utilising physiotherapists whose scope of practice in chronic disease management includes the delivery of multiple interventions.

In this study, there were no statistically significant improvements in patient-reported symptoms or health status scores following the intervention. For example, we observed a mean improvement of 1.35 points on the CAT which was not statistically significant and was below the MCID for PR [37]. However, there is potential that MCIDs for outcomes following PR may not be transferrable to more “light touch” interventions as used in the current study and that a smaller difference would be clinically relevant. The non-significant change observed in some clinical outcomes is consistent with other studies investigating the use of different health professionals for early intervention in the management of people with COPD. For example, Liang et al. (2019) utilised pharmacists for home-based medicines review as well as referral to home-based PR and reported no significant increases in HRQoL or CAT scores at the six and 12 month follow-ups [25]. Similarly, Zwar et al. (2016) focused on upskilling practice nurses to assist in managing COPD within general practices such as supporting smoking cessation, reviewing medications and providing PA and PR recommendations. They also reported similar findings with no significant improvements in HRQoL observed at 12 months [23]. These studies reported low participant attendance rates and poor uptake of the intended interventions by both people with COPD and participating practices as barriers to implementation. Our results were similar to these studies in that there were no significant improvements observed at three months in CAT or mMRC scores, yet uptake of the intervention was significantly better with 78% completing the three-month follow-up assessment.

One possible explanation for the lack of difference in the clinical findings could be due to the close to normal starting point for many outcomes in our group of participants. For example, a large proportion of our sample were classified as ‘new diagnoses’ of COPD and the majority were categorised as GOLD stage I or II with relatively low symptom scores at baseline which could be difficult to significantly improve over a short intervention period. It is likely that more longitudinal data is needed to be able to observe changes in these outcome measures in a population with milder COPD and this is a useful topic of future research. In addition, it has been previously shown that people with the lowest activation levels tend to increase the most following interventions aimed at improving disease management [39]. At baseline, the majority of our cohort reported high health activation with a mean score of 69.2 (Level 4) on the PAM so it is unsurprising that this was difficult to improve further. Conversely, challenges coping with situations such as acquiring a new diagnosis of COPD, could undermine maintenance behaviours and thus correlate with a reduction in health activation score [51]. There is potential that larger effects could have been seen in more moderate to severe cases of COPD with higher symptom scores and lower activation levels and future trials may look to include participants with varied disease severity.

There are several reasons as to why there may have not been a significant change observed in PA outcomes at three months. The fact that this was a brief intervention with minimal contact time between the physiotherapist and participant could offer some explanation. Follow-up appointments with the physiotherapist only occurred at two time points and this amount of contact with the patient may be insufficient to evoke long-term change in PA levels. For example, when comparing our results to studies in COPD also utilising PA counselling and pedometers, some have demonstrated improvements in median step count of up to 1458 steps per day however, many of these studies involved either more frequent follow-up and/or supervision of activity by clinicians [52], highlighting that more frequent intervention, such as attending a PR program, may be necessary to encourage change in PA. An intervention with a higher number of scheduled check-ins should be incorporated into future trials with longer intervention and follow-up periods to examine the impact on participant PA levels in primary care. In addition, research suggests that adults in the “preparation” stage on the PASOCQ are the target population that should be recruited for action-oriented programs and where one would likely observe the most change [53]. The majority of our cohort reported to be in the “maintenance” stage at baseline with already high PA levels indicating little room for further improvement. Interestingly, the majority of participants (4/6) in our study that started in the “preparation” stage moved into the “action” stages at three-months, indicating the potential for some positive change. This study contributes to the current body of literature suggesting that a brief physical activity intervention involving counselling and a pedometer alone in primary care was not sufficient to improve overall physical activity levels in people with COPD with already high activation levels [52].

There were some limitations to this study. Firstly, this was a pilot study with a small sample size receiving the intervention and no comparator group. This small sample size may also have affected the ability to detect significant change in clinical outcomes. Participant recruitment was ceased in January 2020 due to the COVID-19 pandemic which affected our sample size such that the target sample to be screened of 150 to 200 participants was not reached. Incentives or honorariums for practices and patients for participating were not able to be provided and could be considered in future trials to encourage participation and completion. COVID-19 restrictions affected the scheduling of the three-month follow-up assessments and potentially caused higher attrition rates than anticipated. The COVID-19 pandemic may also have impacted on physical activity levels and also PR attendance due to service-related changes during this time in which PR programs in some local health districts were halted. Finally, the sample comprised participants of mostly Caucasian ethnicity with relatively low smoking rates, high education levels and were from a relatively affluent area of Sydney which may limit the generalisability of findings to the wider COPD population. There is also potential that higher numbers of people with COPD may have been recruited if the trial had been conducted in areas with higher smoking rates.

## Conclusion

To our knowledge, this is the first study to examine the impact of a physiotherapist-led intervention where physiotherapists are integrated into primary care to work in partnership with GPs to assist in the management of COPD. The results of this study are consistent with previous research where this model was able to improve some aspects of implementation of COPD management guidelines such as referrals to PR. The potential added value of a physiotherapist who is experienced with COPD management and PR programs, is that they may be more likely to refer to PR and be confident in discussing the benefits of PR with patients, thereby resulting in higher attendance rates. This model did not result in clinical improvements in patient-reported symptom scores, health activation or PA levels in people with COPD, however, other process outcomes such as initiation of smoking cessation interventions and action plans, improved immunisation status, and increased conduct of inhaler technique reviews are encouraging findings. This study is of relevance to service providers and provides useful evidence for policy makers to consider decisions of placement of cardiorespiratory physiotherapists into primary care.

## Supplementary Information

**Additional file 1.** 

## Data Availability

Data will be stored according to and as required by the ethics committee. The data that support the findings of this study are not publicly available and cannot be publicly shared due to ethics requirements. Data are only available from the authors upon reasonable request with permission of the Northern Sydney Local Health District Human Research Ethics Committee. All data and material requests should be addressed to the corresponding author Associate Professor Zoe McKeough or to Professor Sarah Dennis.

## Abbreviations

AAQ: Active Australia Questionnaire
CAT: COPD Assessment Test
CI: Confidence interval
COPD: Chronic Obstructive Pulmonary Disease
FEV_1_: Forced expiratory volume in one second
FVC: Forced vital capacity
GOLD: Global Initiative for Obstructive Lung Disease
GPs: General Practitioners
GPMP: GP management plan
HRQoL: Health-related quality of life
IQR: Interquartile range
LABA: Long-acting beta2-agonists
LAMA: Long acting muscarinic antagonists
MDT: Multidisciplinary team
MID: Minimal important difference
mMRC: Modified Medical Research Council Scale for Dyspnoea
PA: Physical activity
PAM: Patient Activation Measure
PASOCQ: Physical Activity Stages of Change Questionnaire
PR: Pulmonary rehabilitation
QoL: Quality of Life
SABA: Short-acting beta2-agonists
SAMA: Short-acting muscarinic antagonists
SD: Standard deviation
TIDieR: Template for Intervention Description and Replication
