# Supplementary material for: The effects of an innovative GP-physiotherapist partnership in improving COPD management in primary care
Source: BMC Prim Care. 2023 Jul 10;24:142. doi: 10.1186/s12875-023-02097-3 (PMC10332030; doi:10.1186/s12875-023-02097-3)
Supplement: Supplementary file 1 — Additional file 1. [file 12875_2023_2097_MOESM1_ESM.pdf]

Pagano et al. Pilot and Feasibility Studies (2020) 6:138
https://doi.org/10.1186/s40814-020-00680-4

Pilot and Feasibility Studies

STUDY PROTOCOL Open Access

The feasibility of an innovative GP-physiotherapist partnership to identify and manage chronic obstructive pulmonary disease (INTEGRATED): study protocol

Lisa Pagano[1], Zoe McKeough[1], Sally Wootton[1,2], Stephen Crone[2], Deborah Pallavicini[3], Andrew S. L. Chan[4,5], Sriram Mahadev[4,5], Nicholas Zwar[6] and Sarah Dennis[1,7,8,9*]

Abstract

Background: Chronic obstructive pulmonary disease (COPD) contributes significantly to mortality, hospitalisations and health care costs worldwide. There is evidence that the detection, accurate diagnosis and management of COPD are currently suboptimal in primary care. Physiotherapists are well-trained in cardiorespiratory management and chronic care but are currently underutilised in primary care. A cardiorespiratory physiotherapist working in partnership with general practitioners (GPs) has the potential to improve quality of care for people with COPD.

Methods: A prospective pilot study will test the feasibility of an integrated model of care between GPs and physiotherapists to improve the diagnosis and management of people with COPD in primary care. Four general practices will be selected to work in partnership with four physiotherapists from their local health district. Patients at risk of developing COPD or those with a current diagnosis of COPD will be invited to attend a baseline assessment with the physiotherapist, including pre- and post-bronchodilator spirometry to identify new cases of COPD or confirm a current diagnosis and stage of COPD. The intervention for those with COPD will involve the physiotherapist and GP working in partnership to develop and implement a care plan involving the following tailored to patient need: referral to pulmonary rehabilitation (PR), physical activity counselling, medication review, smoking cessation, review of inhaler technique and education. Process outcomes will include the number of people invited and reviewed at the practice, the proportion with a new diagnosis of COPD, the number of patients eligible and referred to PR and the number who attended PR. Patient outcomes will include changes in symptoms, physical activity levels, smoking status and self-reported exacerbations.

Discussion: If feasible, we will test the integration of physiotherapists within the primary care setting in a cluster randomised controlled trial. If the model improves health outcomes for the growing numbers of people with COPD, then it may provide a GP-physiotherapist model of care that could be tested for other chronic conditions.

Trial registration: ANZCTR, ACTRN12619001127190. Registered on 12 August 2019—retrospectively registered.

Keywords: COPD, Primary care, Pulmonary rehabilitation, Physical activity, Screening, Physiotherapy/physical therapy

* Correspondence: sarah.dennis@sydney.edu.au
[1]Discipline of Physiotherapy, University of Sydney, Sydney, Australia
[7]Ingham Institute for Applied Medical Research, Liverpool, Australia
Full list of author information is available at the end of the article

© The Author(s). 2020 **Open Access** This article is licensed under a Creative Commons Attribution 4.0 International License, which permits use, sharing, adaptation, distribution and reproduction in any medium or format, as long as you give appropriate credit to the original author(s) and the source, provide a link to the Creative Commons licence, and indicate if changes were made. The images or other third party material in this article are included in the article's Creative Commons licence, unless indicated otherwise in a credit line to the material. If material is not included in the article's Creative Commons licence and your intended use is not permitted by statutory regulation or exceeds the permitted use, you will need to obtain permission directly from the copyright holder. To view a copy of this licence, visit http://creativecommons.org/licenses/by/4.0/. The Creative Commons Public Domain Dedication waiver (http://creativecommons.org/publicdomain/zero/1.0/) applies to the data made available in this article, unless otherwise stated in a credit line to the data.

Background

Chronic obstructive pulmonary disease (COPD) contributes significantly to mortality, hospitalisations and health care costs worldwide [1]. In Australia alone, COPD contributed to 4.5% of all deaths [2] and was the principal diagnosis for 66,540 hospitalisations for people aged 45 and over in 2015 [3]. COPD is the 11th most commonly managed chronic condition in Australian general practice [4]. The COPD-X Concise Guide for Primary Care identifies general practice as an ideal setting for the diagnosis and management of COPD [5]; however, despite best available evidence-based recommendations, COPD remains under-diagnosed, misdiagnosed and management has been suboptimal in primary care. For example, the Burden of Obstructive Lung Disease Survey found that the prevalence of airflow limitation (GOLD stage II or higher) in Australians 40 years and over was 7.5%, whereas the prevalence of doctor-diagnosed COPD in the same population was only 5.2% [6].

High-quality spirometry testing is one way to improve the diagnosis of COPD in primary care [5, 7] yet evidence indicates that the use of spirometry in general practice is low [8–10]. There are multiple factors contributing to the suboptimal use of spirometry in primary care. Firstly, GPs are not adequately reimbursed by Medicare to undertake pre- and post-bronchodilator spirometry with current reimbursement being only $34.95 from the Medicare Benefits Schedule which can only be claimed once in any 12-month period. A further barrier is that some GPs and practice nurses (PNs) lack confidence in the interpretation of spirometry [11] with one study reporting that GPs and PNs misinterpreted spirometry in 30% of cases [12].

Improvement in the diagnosis of COPD could be achieved through case-finding methods. This involves the identification of 'high-risk patients' and utilising standardised screening questionnaires in addition to spirometry. Previous studies have targeted people over the age of 40 years who are current or ex-smokers with a 10-year or more pack history and have focused on upskilling GPs, PNs or research assistants at performing these screening techniques. These methods have been shown to be effective with the identification of new cases of COPD ranging from 4 to 27%, as well as identifying a high rate of misdiagnosis [12–14].

Once COPD has been confirmed and pharmacological management optimised, one of the most effective non-pharmacological interventions for individuals with COPD is pulmonary rehabilitation (PR). PR has been shown to lead to improvements in exercise tolerance, dyspnoea, depression and overall health-related quality of life [15–18]. However, despite strong international recommendations, referral to PR from healthcare professionals is low. It is estimated that only 3–16% of eligible patients are referred to PR and even fewer people (1–2%) actually receive this intervention [16, 19]. In particular, referral rates from primary care are low with one study reporting only 5% of eligible patients were referred to PR by general practitioners [20]. The barriers to referral and attendance at PR are well known. These include low awareness and knowledge of PR and the benefits of PR, low knowledge of the referral process and a lack of persuasive health professional communication [21–23]. Current guidelines also recommend the use of multidisciplinary care plans in the management of patients with COPD [5]. Multidisciplinary care plans, initiated by GPs, anticipate the wide range of long-term needs of patients with COPD and have been found to improve exercise capacity and health-related quality of life and reduce hospitalisation [24–26]. However, some studies have found that GPs need more support to develop and implement multidisciplinary care plans in some chronic conditions such as asthma [27, 28].

The issues surrounding the management of COPD in primary care are complex, and there is a need for alternative strategies to improve outcomes for patients with COPD. Zwar et al. (2016) tested a GP-nurse partnership approach in the management of newly diagnosed COPD and found no difference in outcomes compared to usual care [12]. Furthermore, they found that some PNs still lacked confidence in the diagnosis and management of COPD despite additional training [11]. One alternative method could be to utilise multi-component interdisciplinary management which, in a COPD population, has shown improvements in some patient outcomes including disease-specific quality of life [29, 30], patient self-reported physical activity levels [31] and exercise capacity [30]. A Cochrane review has reported a reduction in hospital admissions and hospital days per person in favour of the inter-disciplinary management approach [30].

Physiotherapists play an important role in respiratory disease management in Australian hospitals, particularly in coordinating PR programmes and in providing physical activity advice. Physiotherapy students in Australia undertake extensive training in cardiopulmonary physiotherapy. This training includes the use and interpretation of spirometry, conducting exercise testing for people with respiratory disease and exercise prescription within a PR programme. Physiotherapists are, therefore, well positioned to work in partnership with GPs to better manage patients with chronic lung disease but are currently underused in this role in primary care. Physiotherapy services, as a first point of contact, have been shown to be safe, effective and well received by patients with musculoskeletal complaints [32, 33]. There is currently no published literature on a GP-physiotherapist partnership model in primary care in an Australian setting to improve identification and management of COPD.

Study aims and hypothesis

The primary aim of the INTEGRATED study (InNovaTivE Gp-physiotheRapist pArTnErship for copD) is to determine the feasibility and acceptability of this integrated model of GP and physiotherapist partnership. The secondary aims are to (1) produce high-quality spirometry with accurate interpretation to both identify new cases of COPD and determine the stage of severity for known cases of COPD, (2) increase general practice referrals to pulmonary rehabilitation for people that meet the COPD-X guidelines criteria for referral [5], (3) increase physical activity levels in people with COPD at 3 months compared to baseline and (4) increase smoking cessation in those who are smokers at baseline.

The INTEGRATED study hypothesises that a GP and physiotherapist model of care will be a feasible and acceptable way to improve the diagnosis and management of people with COPD. This will be achieved through more accurate spirometry interpretation, at least 90% of participants who meet the COPD-X guidelines for referral to PR being referred to a programme, an increase in physical activity levels by participants at 3 months compared to baseline, and at least 50–75% of participants have attended or commenced a smoking cessation programme at 3 months after their baseline visit.

Methods

The INTEGRATED study is a before and after feasibility study within the 'health services' research domain with the aim of trialling a new model of care intended for use in a larger cluster randomised controlled trial (RCT). The protocol has followed the TIDieR (Template for Intervention Description and Replication framework) Framework. The study is being conducted in metropolitan Sydney, Australia, and commenced recruitment in October 2018 with the aim of continuing to review participants into June 2020. The research and data collection plan is summarised in Fig. 1. A minimum of four general practices will be recruited to the study from a local Primary Health Network (PHN). Physiotherapists from the rehabilitation service of the corresponding Local Health District (LHD) will run a weekly respiratory clinic at each of the participating general practices.

Inclusion criteria

General practices

General practices will be eligible if they use computerised clinical software, have a room available for use by the physiotherapist and are interested in an integrated GP-physiotherapist partnership model for COPD management. Where possible, general practices will be chosen that meet the PHN strategic directions for integrated care and are close to one of the PR programmes provided by the LHD.

Patients

The INTEGRATED study will target two groups of patients—new cases of COPD and those with existing COPD. The inclusion criteria have been successfully used in a previous primary care COPD case-finding study [12].

New cases of COPD

Patients will be eligible if they have attended the practice at least twice with one visit in the preceding 12 months, are aged 40 years or over and have a documented history of smoking (current or former smoker) in their medical notes. A history of current smoking or past smoking will identify those patients who are at high risk of developing COPD.

Existing COPD

Patients will be eligible if they are aged 40 years or over, have a recorded diagnosis of COPD or are taking medications prescribed for COPD (such as short-acting inhaled β_2 agonists (SABA), short-acting muscarinic antagonists (SAMA), long-acting inhaled β_2 agonists (LABA), long-acting muscarinic antagonists (LAMA), combination of LABA/LAMA and inhaled corticosteroids), and they have visited the practice in the last 12 months.

Patients will be excluded if they have terminal cancer, cognitive impairment, require home oxygen, do not speak sufficient English or are pregnant.

Recruitment

General practice and physiotherapist recruitment

Recruitment will be conducted with assistance from the PHN. An expression of interest will be included in the newsletter sent to local practices. Those practices expressing an interest in taking part in the study will be visited by an investigator to discuss the study and answer any questions. The practices will be provided with an information sheet and asked to provide written consent to participate in the study. As requested by the local ethics committee, a contract will be prepared and signed between the LHD and the general practices taking part in the study. This will be forwarded to the ethics committee before the practice starts the study. The LHD will identify a senior respiratory physiotherapist with at least 5 years of clinical experience in the management of chronic respiratory conditions to partner with each of the participating general practices. These physiotherapists are experienced senior clinicians who have specialist knowledge within their discipline [34] and have undergone extensive training to develop their skill set for chronic respiratory disease management. They will have high-level skills in spirometry including interpretation, COPD management and self-management, exercise prescription and physical activity advice.

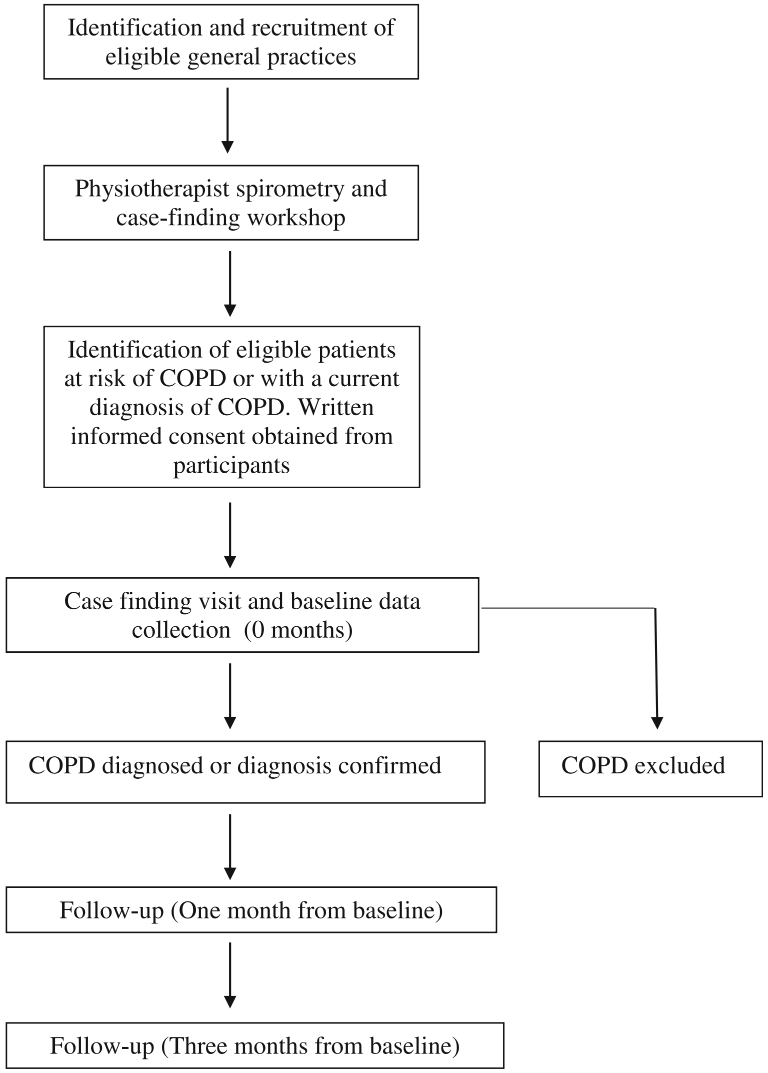

Fig. 1 Study and data collection processes

Training of physiotherapists

All physiotherapists will receive training in case-finding for the diagnosis of COPD. This will comprise two workshops. This first workshop will cover the case-finding approach for the diagnosis of COPD with performance of spirometry based on the European Respiratory Society (ERS)/American Thoracic Society (ATS) quality criteria and its interpretation [35] as this is essential to the accurate diagnosis and assessment of COPD [5]. The second workshop will include training on COPD management such as smoking cessation advice and referral, physical activity advice and referral to pulmonary rehabilitation if required, preparation of a care plan with the general practice team and the processes for the case-finding and follow-up appointments.

Patient recruitment

Potentially eligible patients will be identified from a search of the practice electronic records of all participating general practices by either a research assistant or trained practice staff. The resultant list will be checked for exclusions by the GPs and /or PNs. All potentially eligible patients will be sent an invitation from the practice inviting them to take part in the study. Those interested in the study will be asked to return the completed form in a pre-paid envelope and to also provide written informed consent.

Patient and public involvement statement

Patient input was obtained from the qualitative feedback of the PELICAN study [12] and was used to refine the

model of care to determine the added value of the physiotherapist to the primary care team. It was not possible to involve patients in the study design beyond this. The qualitative feedback from the participants in this pilot study will be used to further modify the intervention for a larger study. We had health service involvement in the setting of the study aims from the rehabilitation service in the LHD. They were directly involved in establishing the project around the priorities for their chronic disease integration service which aimed to engage with a less severe patient population in primary care who would benefit from pulmonary rehabilitation.

Study processes

Baseline assessment

All patients (new cases and existing COPD) will be required to complete the following questionnaires at baseline: demographic questionnaire, COPD Diagnostic Questionnaire (CDQ) [36], COPD Assessment Test (CAT) [37], Modified Medical Research Council Dyspnoea Scale (mMRC) [38], Patient Activation Measure (PAM) [39, 40], Physical Activity Stages of Change Questionnaire (PASOCQ) [41] and Active Australia Questionnaire (AAQ) [42]. All patients will undergo pre- and post-bronchodilator spirometry based on the ERS/ATS lung function guidelines [35]. The physiotherapist will then determine the level of obstruction for those with existing COPD or determine whether the patient has a diagnosis of COPD which will be based on post-bronchodilator results of a forced expiratory volume in one second (FEV_1)/forced vital capacity (FVC) < 0.7. Patients will be included for follow-up testing in the study based on a COPD diagnosis from spirometry testing determined by the physiotherapist. If spirometry appears abnormal for other reasons, the results will be discussed with the GP and where appropriate patients will be referred for further testing with respiratory specialists.

Intervention

The intervention will be coordinated by the physiotherapist at each site under the supervision of the general practice staff. Participants will be free to decline or discontinue any or all of the intervention components at any point in time.

New cases of COPD

The physiotherapist will initiate and work in partnership with the GP and patient to develop a COPD specific GP Management Plan (GPMP) and where necessary, Team Care Arrangement (TCA) [43] for the participant using the template developed from the PELICAN Study [44]. This is necessary in Australia to access Medicare subsidised management by an allied health provider such as a physiotherapist. The problems identified in the assessment and the plan of action including details of specific management will be documented. If necessary, patients will also be referred back to the GP for review and medical management as required. Patients will receive physical activity advice and counselling using the 5 A's approach (Ask, Advise, Assess, Assist, Arrange follow-up) according to the Australian Physical Activity and Sedentary Behaviour Guidelines [45] and a pedometer to monitor their physical activity goals and guide exercise prescription at follow-up appointments. The 5As are a clinical tool recommended for health behaviour counselling in primary care which will enable the physiotherapist to identify barriers to engaging in regular physical activity as well as strategies to overcome these barriers and tailor physical activity goals towards the participant. Patients will also be referred to PR if they meet the requirement according to the COPD-X guidelines [5]. These guidelines state that all patients with COPD (of all mMRC grades) will benefit from PR and recommend that all patients with exertional dyspnoea, those at risk of exacerbation and following an exacerbation should be referred to PR [5]. All current smokers will receive individualised smoking cessation advice and support and appropriate referrals will be made back to the GP as informed by the Royal Australian College of General Practitioners (RACGP) guidelines [46]. Participant education booklets regarding physical activity guidelines, smoking cessation and COPD management will also be provided.

Existing COPD

As for new cases of COPD, the physiotherapist will review the current GPMP and TCA or develop one in partnership with the GP if there is no existing GPMP/TCA. Patients with existing COPD will also receive physical activity advice and counselling using the 5 A's approach, a pedometer and smoking cessation advice and referral if applicable. Patients will also be referred to PR if they meet the requirement according to the COPD-X guidelines [5] and receive participant education booklets regarding physical activity guidelines, smoking cessation and COPD management.

Those without COPD on spirometry

Physical activity advice and counselling using the 5 A's approach will be provided according to the Australian Physical Activity and Sedentary Behaviour Guidelines [45]. All current smokers will receive individualised smoking cessation advice and support and appropriate referrals will be made back to the GP. These patients will not return for further follow-up with the physiotherapist.

Follow-up assessment

All participants with existing or newly diagnosed COPD will return for a follow-up visit with the physiotherapist at 1 month to review current physical activity levels and establish a physical activity goal to progress towards at 3 months. A final assessment will be conducted at 3 months to review physical activity goals and complete questionnaires.

Outcome measures

The physiotherapist will collect demographic information including age, gender, body mass index, employment status, education and country of birth for all individuals who attend the respiratory clinic. Patient and process outcomes will also be collected in people with existing COPD and those with newly diagnosed COPD.

Data will be collected at baseline and at the 1-month and 3-month follow-up visits so the level of change in the outcomes following the intervention can be determined. If patients are unable to attend the appointment at 3 months with the physiotherapist, the research assistant will complete the assessment by telephone.

Primary outcomes

Feasibility outcomes A summary of the outcomes that will be used to determine the feasibility of this health service model on the effective identification and management of COPD can be found in Table 1. The primary outcome measures will be number (%) identified with obstruction from the case-finding cohort and number (%) eligible and referred to PR programmes. The success target for number (%) identified with obstruction will be set at 15% as previous studies have reported rates of case finding new diagnoses of COPD between 4 and 27% [12–14]. International literature suggests that only 5% of eligible patients are referred by GPs to PR [20]. We would determine the clinics successful if 80% of eligible participants are referred to PR by the clinics and at least 50% of those referred attend an appointment.

Acceptability of the partnership approach All the participating GPs and physiotherapists will be asked to take part in one semi-structured interview to explore their experiences of providing an integrated model of care for COPD. There will be at least four GPs and four physiotherapists interviewed. A sample of patients (n = 20) will also be asked to take part in semi-structured interviews to examine their experience of being involved in the INTEGRATED study. Themes explored will include satisfaction with the programme and the effects and value of physiotherapist input into COPD diagnosis and management. Barriers and facilitators to the integrated management approach, as well as to access and uptake of evidence-based interventions such as PR, will also be examined. GPs and physiotherapists will be asked to reflect on their roles in the project and how they worked together to manage people with COPD. Physiotherapists will also be asked to reflect on their skill set and how their skill set prepared them to be integrated into the general practice team. All interviews will be digitally recorded and transcribed verbatim in preparation for thematic analysis.

Secondary outcomes The secondary outcome measures are summarised in Table 2 and consist of the patient-relevant outcomes. Health status and symptom score will be measured by the CAT. The CAT has been shown to be a validated tool in the assessment of disease-related quality of life [37] with the most reliable estimate of the minimum important difference being a change of 2 points [47]. Dyspnoea will be assessed using the

Table 1 Summary of process outcomes to determine feasibility

Existing COPD	New cases of COPD
Number (%) with spirometry completed (FEV_1 and FVC data) meeting ERS/ATS quality criteria	Number (%) with spirometry completed (FEV_1 and FVC data) meeting ERS/ATS quality criteria
Number of known COPD patients over 40 years in the general practice	Number of smokers/ex-smokers over 40 years in the general practice
Number (%) invited to attend	Number (%) invited to attend
Number (%) attended first appointment with physiotherapist	Number (%) attended first appointment with physiotherapist
Number (%) confirmed diagnosis and severity	Number (%) confirmed diagnosis and severity
Number (%) eligible for PR	Number (%) eligible for PR
Number (%) referred to PR	Number (%) referred to PR
Number (%) attended PR	Number (%) attended PR

Table 2 Summary of patient outcomes

Existing COPD	New cases of COPD
Symptom Score (CAT)	Symptom Score (CAT)
Meeting physical activity guidelines (Y/N) self-reported using AAQ	Meeting physical activity guidelines (Y/N) self-reported using AAQ
COPD Diagnostic Questionnaire (CDQ) score	COPD Diagnostic Questionnaire (CDQ) score
Dyspnoea score (mMRC)	Dyspnoea score (mMRC)
Patient Activation Measure (PAM)	Patient Activation Measure (PAM)
Daily step count (pedometer)	Daily step count (pedometer)
Smoking status	Smoking status
Self-reported exacerbations	Self-reported exacerbations
Self-reported hospital admission	Self-reported hospital admission
Self-reported emergency attendance	Self-reported emergency attendance

mMRC [38] which is rated on a scale of 0 ('I only get breathless with strenuous exercise') to 4 ('I am too breathless to leave the house' or 'I am breathless when dressing or undressing').

Physical activity will be measured in two ways. The first method will be self-reported activity levels and whether they are meeting the Australian Physical Activity and Sedentary Behaviour Guidelines [45] determined by the AAQ. The second method is the recording of daily steps from a pedometer which will be worn either around the patient's neck or in their pocket. To assist the physiotherapist in exercise prescription, the PASOCQ [41] will also be used to determine the participant's readiness for change. This questionnaire consists of 4 questions which places participant's into one of the five stages of change (pre-contemplation, contemplation, preparation, action and maintenance phases).

Patient health activation will be assessed using the PAM [40] which is a 22-item measure that assesses patient knowledge, skill and confidence for self-management.

Hospital utilisation will be assessed through self-report of exacerbations, hospital admissions and emergency department attendance.

The CDQ [36] will also be assessed which is an 8-item tool developed to identify those who would benefit from spirometry testing to avoid spending time conducting tests on those unlikely to test positive. The sum score of the CDQ ranges from 0 to 38 and divides subjects into three groups of COPD likelihood: low (< 16.5), medium (16.5–19.5) and high (> 19.5).

Sample size and feasibility We will recruit a minimum of four general practices to the study. Based on data from a previous study [48], it is anticipated that each practice will identify 104 potentially eligible patients with a diagnosis of COPD with 50% participation and 52 patients are likely to consent to take part in the study. It is estimated that the four practices will identify a total of 1060 patients who are at risk of COPD but without a current diagnosis. Of these, 17% (180) will attend for spirometry and assessment and 18% [33] will receive a new diagnosis of COPD [12].

Statistical analysis

Data will be analysed using SPSS statistical software with a two-sided significance level set to 0.05. Descriptive analyses will be used to evaluate the process outcomes. Paired *t* test for significance will be performed to compare patient responses in the baseline and 3-month follow-up questionnaires. Interviews with the participating GPs, PNs and physiotherapists will be undertaken and will be digitally recorded, transcribed verbatim and analysed thematically. The researchers will code the data using NVivo. We will use the Theoretical Domains Framework to guide the coding and analysis of the health professional transcripts [49]. The patient transcripts will be coded thematically and triangulated with the health professional data.

Discussion

We anticipate that the GP and physiotherapist partnership will be feasible and will improve the diagnosis and management of COPD in primary care in the participating practices. Current evidence suggests that spirometry use in primary care is low [8–10] and misinterpretation of spirometry results and misdiagnosis of COPD is high [12–14]. Lack of confidence expressed by GPs and PNs at interpreting spirometry [11] could contribute to this, and there is also recent evidence emerging regarding people with COPD fluctuating in and out of diagnostic status. One study [50] found that up to one-third of symptomatic ex-smokers with baseline obstruction on diagnostic spirometry had shifted to non-obstructed when routinely re-tested after 1 or 2 years. This highlights the difficulties surrounding diagnosis and the importance of follow-up testing. We hope that including physiotherapists with expertise in the performance and interpretation of spirometry will improve the accuracy of diagnosis as well as identify new cases of COPD in those deemed 'at risk' of developing COPD. Studies have also reported suboptimal referral by GPs to pulmonary rehabilitation and poor patient attendance and completion rates [20–23, 51, 52] which is one of the most effective treatments for COPD. More effective management of COPD in primary care and increased uptake of pulmonary rehabilitation could lead to improvements in the quality of care and health outcomes for people with COPD.

In Australia, physiotherapists are highly trained in the management of cardiorespiratory problems in secondary care. Australian physiotherapists are crucial to the delivery of exercise training programmes including PR, as well as providing therapies to increase sputum production, reduce work of breathing and improve overall ventilation. Respiratory physiotherapists in Europe also undertake extensive training in these areas as well as conducting and interpreting pulmonary function testing [53]. Physiotherapy services, as a first point of contact, have been shown to be effective in patients with musculoskeletal skeletal complaints [32] and a viable alternative to GP care. It was suggested in another study [33] that up to 85% of musculoskeletal complaints in a general practice in Sweden could be managed solely by the physiotherapist. Currently, there is no published research on the effectiveness of physiotherapists providing cardiorespiratory

interventions in partnership with GPs and this model of care needs further investigation within an Australian landscape.

The INTEGRATED study will test the efficacy of a new model of care integrating experienced cardiorespiratory physiotherapists with GPs to improve outcomes for people with COPD. Proven case-finding methods in an at-risk population will be used to identify new cases of COPD and confirm current diagnoses, as well as implement evidence-based guidelines for COPD management. This study targets COPD patients within a primary care setting where the contact with potential COPD patients with mild to moderate obstruction is greatest and where improved care has the potential for substantial health benefit. The process outcome measures that will be used in this study are based on components of evidence-based care for patients with COPD [5]. These measures will allow us to determine the integrity of the intervention and to what extent the physiotherapist was able to support the GP in delivering best practice. Other outcome measures used will enable us to measure the impact of the care provided on patient-level outcomes for example impact on health status, symptom scores, smoking status and self-reported hospital utilisation. The results from the qualitative interviews, particularly feedback obtained from participants in this study, will be used to inform and further modify the intervention for future randomised controlled trials (RCTs). A limitation to this study is that assessment measures will be performed by the treating physiotherapist and not a blinded assessor. Whilst many of the outcomes assessed in this study are self-reported which reduces the impact of bias, any future RCTs will have blinded assessors.

If this model of GP physiotherapist partnership is feasible, further research will test the effectiveness and cost-effectiveness of this model in a RCT. This model has the potential to inform models of care that better integrate physiotherapists (upskilled in cardiothoracic physiotherapy) with GPs to improve service delivery to the increasing numbers of people with COPD in Australia. Furthermore, there are several other chronic conditions such as osteoarthritis, other musculoskeletal conditions and falls that are commonly encountered in primary care with effective physiotherapy interventions. This model of care for COPD could provide a template for integrating physiotherapists more effectively into the general practice team to inform other models of care and improve outcomes for people with these chronic conditions. Currently, there is no research evidence for physiotherapists and GPs working in partnership in this way in Australia so this project will provide the first examination of this integrated care approach.

Supplementary information

Supplementary information accompanies this paper at https://doi.org/10.1186/s40814-020-00680-4.

Additional file 1. The TIDieR (Template for Intervention Description and Replication) Checklist: This additional file provides a checklist of the TIDieR framework and demonstrates how this protocol adheres to it.

Abbreviations

AAQ: Active Australia Questionnaire; ATS: American Thoracic Society; CAT: COPD Assessment Test; COPD: Chronic obstructive pulmonary disease; CDQ: COPD Diagnostic Questionnaire; ERS: European Respiratory Society; FEV_1: Forced expiratory volume in one second; FVC: Forced vital capacity; GPs: General practitioners; GPMP: GP management plan; LABA: Long-acting beta2-agonists; LAMA: Long-acting muscarinic antagonists; LHD: Local Health District; mMRC: Modified Medical Research Council Scale for Dyspnoea; PAM: Patient Activation Measure; PASOCQ: Physical Activity Stages of Change Questionnaire; PHN: Primary Health Network; PNs: Practice nurses; PR: Pulmonary rehabilitation; RCT: Randomised controlled trial; RCTs: Randomised controlled trials; SABA: Short-acting beta2-agonists; SAMA: Short-acting muscarinic antagonists; TCA: Team care arrangement; TIDieR: Template for Intervention Description and Replication

Acknowledgements

The authors would like to acknowledge the contributions of the Northern Sydney Local Health District Chronic Disease Community Rehabilitation Service, Sydney North Primary Health Network and all the general practices that have agreed to participate.

Authors' contributions

The study was conceived by ZM, SW and SD and all authors contributed to the study design. LP wrote the initial draft of the manuscript. All authors contributed to and approved the final version of the manuscript.

Funding

This project was funded from a small grant from the Chronic and Complex Care, Primary and Community Health, Northern Sydney Local Health District. The content is solely the responsibility of the authors and does not necessarily represent the official view of the NSLHD.

Availability of data and materials

Data will be stored according as required by the ethics committee and will be available from the authors on request.

Ethics approval and consent to participate

This study has been approved by the Northern Sydney Local Health District Human Research Ethics committee (HREC reference; HREC/15/HAWKE/434). All participants will give their written informed consent to participate in the study.

Consent for publication

Not applicable

Competing interests

The authors declare that they have no competing interests.

Author details

[1]Discipline of Physiotherapy, University of Sydney, Sydney, Australia. [2]Chronic Disease Community Rehabilitation Service, Northern Sydney Local Health District, St Leonards, Australia. [3]Sydney North Primary Health Network (SNPHN), St Leonards, Australia. [4]Royal North Shore Hospital, St Leonards, Australia. [5]Northern Clinical School, University of Sydney, Sydney, Australia. [6]Faculty of Health Sciences and Medicine, Bond University, Gold Coast, Australia. [7]Ingham Institute for Applied Medical Research, Liverpool, Australia. [8]South Western Sydney Local Health District, Liverpool, Australia. [9]Faculty of Health Sciences, The University of Sydney, 75 East Street, Lidcombe, NSW 2141, Australia.

Received: 2 January 2020 Accepted: 8 September 2020
Published online: 23 September 2020

References

1. World Health Organisation. World Health Statistics. Geneva: World Health Organisation; 2008.
2. Australian Institute of Health and Welfare. Potentially preventable hospitalisations in Australia by small geographic area. Canberra: AIHW; 2018.
3. Australian Institute of Health and Welfare. Australian Burden of Disease Study: impact and causes of illness and death in Australia 2011; 2016. p. 287.
4. Britt H, Miller GC, Henderson J, Bayram C, Valenti L, Harrison C, et al. A decade of Australian general practice activity 2004–05 to 2013–14. Sydney: Sydney University Press; 2014.
5. Yang IA, Brown JL, George J, et al. COPD-X Australian and New Zealand guidelines for the diagnosis and management of chronic obstructive pulmonary disease: 2017 update. Med J Aust. 2017;207(10):436–42. https://doi.org/10.5694/mja17.00686.
6. Toelle BG, Xuan W, Bird TE, Abramson MJ, Atkinson DN, Burton DL, et al. Respiratory symptoms and illness in older Australians: the Burden of Obstructive Lung Disease (BOLD) study. Med J Aust. 2013;198(3):144–8.
7. Global Initiative for Chronic Obstructive Lung Disease. Global strategy for diagnosis, management, and prevention of chronic obstructive pulmonary disease (2019 Report). 2019. https://goldcopd.org/wp-content/uploads/2018/11/GOLD-2019-v1.7-FINAL-14Nov2018-WMS.pdf.
8. Walters JA, Hansen EC, Johns DP, Blizzard EL, Walters EH, Wood-Baker R. A mixed methods study to compare models of spirometry delivery in primary care for patients at risk of COPD. Thorax. 2008;63(5):408–14.
9. Miravitlles M, de la Roza C, Naberan K, Lamban M, Gobartt E, Martin A. Use of spirometry and patterns of prescribing in COPD in primary care. Respir Med. 2007;101(8):1753–60.
10. Joo MJ, Au DH, Fitzgibbon ML, McKell J, Lee TA. Determinants of spirometry use and accuracy of COPD diagnosis in primary care. J Gen Intern Med. 2011;26(11):1272.
11. Dennis S, Reddel HK, Middleton S, Hasan I, Hermiz O, Phillips R, et al. Barriers and outcomes of an evidence-based approach to diagnosis and management of chronic obstructive pulmonary disease (COPD) in Australia: a qualitative study. Fam Pract. 2017;34(4):485–90.
12. Zwar NA, Bunker JM, Reddel HK, Dennis SM, Middleton S, van Schayck OC, et al. Early intervention for chronic obstructive pulmonary disease by practice nurse and GP teams: a cluster randomized trial. Fam Pract. 2016;33(6):663–70.
13. Bunker J, Hermiz O, Zwar N, Dennis SM, Vagholkar S, Crockett A, et al. Feasibility and efficacy of COPD case finding by practice nurses. Aust Fam Physician. 2009;38(10):826–30.
14. Van Schayck C, Loozen J, Wagena E, Akkermans R, Wesseling G. Detecting patients at a high risk of developing chronic obstructive pulmonary disease in general practice: cross sectional case finding study. BMJ. 2002;324(7350):1370.
15. Vestbo J, Hurd SS, Agustí AG, Jones PW, Vogelmeier C, Anzueto A, et al. Global strategy for the diagnosis, management, and prevention of chronic obstructive pulmonary disease: GOLD executive summary. Am J Respir Crit Care Med. 2013;187(4):347–65.
16. McCarthy B, Casey D, Devane D, Murphy K, Murphy E, Lacasse Y. Pulmonary rehabilitation for chronic obstructive pulmonary disease. Cochrane Database Syst Rev. 2015;2:CD003793.
17. Troosters T, Casaburi R, Gosselink R, Decramer M. Pulmonary rehabilitation in chronic obstructive pulmonary disease. Am J Respir Crit Care Med. 2005;172(1):19–38.
18. Ries AL, Bauldoff GS, Carlin BW, Casaburi R, Emery CF, Mahler DA, et al. Pulmonary rehabilitation: joint ACCP/AACVPR evidence-based clinical practice guidelines. Chest. 2007;131(5):4S–42S.
19. Johnston K, Grimmer-Somers K. Pulmonary rehabilitation: overwhelming evidence but lost in translation? Physiother Can. 2010;62(4):368–73.
20. Perez X, Wisnivesky JP, Lurslurchachai L, Kleinman LC, Kronish IM. Barriers to adherence to COPD guidelines among primary care providers. Respir Med. 2012;106(3):374–81.
21. Milner SC, Boruff JT, Beaurepaire C, Ahmed S, Janaudis-Ferreira T. Rate of, and barriers and enablers to, pulmonary rehabilitation referral in COPD: a systematic scoping review. Respir Med. 2018;137:103–14.
22. Johnston KN, Young M, Grimmer-Somers KA, Antic R, Frith PA. Why are some evidence-based care recommendations in chronic obstructive pulmonary disease better implemented than others? Perspectives of medical practitioners. Int J Chron Obstruct Pulmon Dis. 2011;6:659–67.
23. Mathar H, Fastholm P, Hansen IR, Larsen NS. Why do patients with COPD decline rehabilitation. Scand J Caring Sci. 2016;30(3):432–41.
24. Lorig KR, Sobel DS, Stewart AL, et al. Evidence suggesting that a chronic disease self-management program can improve health status while reducing hospitalization: a randomized trial. Med Care. 1999;37(1):5–14. https://doi.org/10.1097/00005650-199901000-00003.
25. Peytremann-Bridevaux I, Staeger P, Bridevaux P-O, Ghali WA, Burnand B. Effectiveness of chronic obstructive pulmonary disease-management programs: systematic review and meta-analysis. Am J Med. 2008;121(5):433–43 e4.
26. Chavannesa NH, Grijsen M, van den Akker M, Schepers H, Nijdam M, Tiep B, et al. Integrated disease management improves one-year quality of life in primary care COPD patients: a controlled clinical trial. Prim Care Respir J. 2009;18(3):171.
27. Blakeman TM, Comino EJ, Zwar NA, Harris MF. Evaluating general practitioners' views about the implementation of the Enhanced Primary Care Medicare items. Med J Aust. 2001;175(2):95–8.
28. Zwar NA, Hasan I, Comino EJ, Harris MF. General practitioner views on barriers and facilitators to implementation of the Asthma 3+ Visit Plan. Med J Aust. 2005;183(2):64–7.
29. Liang J, Abramson MJ, Russell G, Holland AE, Zwar NA, Bonevski B, et al. Interdisciplinary COPD intervention in primary care: a cluster randomised controlled trial. Eur Respir J. 2019.
30. Kruis AL, Smidt N, Assendelft WJ, Gussekloo J, Boland MR, Rutten-van Mölken M, et al. Integrated disease management interventions for patients with chronic obstructive pulmonary disease. Cochrane Database Syst Rev. 2013;10.
31. Kruis AL, Boland MR, Assendelft WJ, Gussekloo J, Tsiachristas A, Stijnen T, et al. Effectiveness of integrated disease management for primary care chronic obstructive pulmonary disease patients: results of cluster randomised trial. BMJ. 2014;349:g5392.
32. Goodwin RW, Hendrick PA. Physiotherapy as a first point of contact in general practice: a solution to a growing problem? Prim Health Care Res Dev. 2016;17(5):489–502.
33. Ludvigsson ML, Enthoven P. Evaluation of physiotherapists as primary assessors of patients with musculoskeletal disorders seeking primary health care. Physiotherapy. 2012;98(2):131–7.
34. NSW IRCo. NSW Health Service Health Professionals (State) Award 2019 2019. Available from: https://www.health.nsw.gov.au/careers/conditions/Awards/health-professional.pdf.
35. Miller MR, Hankinson J, Brusasco V, Burgos F, Casaburi R, Coates A, et al. Standardisation of spirometry. Eur Respir J. 2005;26:319–38.
36. Price DB, Tinkelman DG, Nordyke RJ, Isonaka S, Halbert R. Scoring system and clinical application of COPD diagnostic questionnaires. Chest. 2006;129(6):1531–9.
37. Jones P, Harding G, Berry P, Wiklund I, Chen W, Leidy NK. Development and first validation of the COPD Assessment Test. Eur Respir J. 2009;34(3):648–54.
38. Mahler DA, Wells CK. Evaluation of clinical methods for rating dyspnea. Chest. 1988;93(3):580–6.
39. Hibbard JH, Stockard J, Mahoney ER, Tusler M. Development of the Patient Activation Measure (PAM): conceptualizing and measuring activation in patients and consumers. Health Serv Res. 2004;39(4p1):1005–26.
40. Hibbard JH, Mahoney ER, Stockard J, Tusler M. Development and testing of a short form of the patient activation measure. Health Serv Res. 2005;40(6p1):1918–30.
41. Marcus B, Rossi J, Selby V, Niaura R, Abrams D, Marcus B. The stages and processes of exercise adoption and maintenance in a worksite sample. Health Psychol. 1992;11(6):386–95.
42. Australian Institute of Health and Welfare. The Active Australia Survey: a guide and manual for implementation, analysis and reporting. Canberra: AIHW; 2003.
43. The Department of Health. Chronic disease management patient information [updated 14 March 2014. Available from: https://www.health.gov.au/internet/main/publishing.nsf/Content/mbsprimarycare-chronicdisease-pdf-infosheet.
44. Bunker JM, Reddel HK, Dennis SM, Middleton S, Van Schayck C, Crockett AJ, et al. A pragmatic cluster randomized controlled trial of early intervention for chronic obstructive pulmonary disease by practice nurse-general practitioner teams: Study Protocol. Implement Sci. 2012;7(1):83.

45. The Department of Health. Australia's physical activity and sedentary behaviour guidelines and the Australian 24-hour movement guidelines: The Australian Government, Department of Health; [updated 12 April 2019. Available from: https://www.health.gov.au/internet/main/publishing.nsf/Content/health-pubhlth-strateg-phys-act-guidelines.
46. The Royal Australian College of General Practitioners. Supporting smoking cessation: A guide for health professionals. 2nd edn. East Melbourne, Vic: RACGP; 2019.
47. Kon SS, Canavan JL, Jones SE, Nolan CM, Clark AL, Dickson MJ, et al. Minimum clinically important difference for the COPD Assessment Test: a prospective analysis. Lancet Respir Med. 2014;2(3):195–203.
48. Zwar NA, Hermiz O, Comino E, Middleton S, Vagholkar S, Xuan W, et al. Care of patients with a diagnosis of chronic obstructive pulmonary disease: a cluster randomised controlled trial. Med J Aust. 2012;197(7):394–8.
49. Cane J, O'Connor D, Michie S. Validation of the theoretical domains framework for use in behaviour change and implementation research. Implement Sci. 2012;7(1):37.
50. Schermer TR, Robberts B, Crockett AJ, Thoonen BP, Lucas A, Grootens J, et al. Should the diagnosis of COPD be based on a single spirometry test? NPJ Prim Care Respir Med. 2016;26:16059.
51. Ta M, George J. Management of chronic obstructive pulmonary disease in Australia after the publication of national guidelines. Intern Med J. 2011;41(3):263–70.
52. Bourbeau J, Sebaldt RJ, Day A, Bouchard J, Kaplan A, Hernandez P, et al. Practice patterns in the management of chronic obstructive pulmonary disease in primary practice: the CAGE study. Can Respir J. 2008;15(1):13–9.
53. Troosters T, Tabin N, Langer D, Burtin C, Chatwin M, Clini EM, et al. Introduction of the harmonised respiratory physiotherapy curriculum. Breathe. 2019;15(2):110–5.

Publisher's Note

Springer Nature remains neutral with regard to jurisdictional claims in published maps and institutional affiliations.

Ready to submit your research? Choose BMC and benefit from:

- fast, convenient online submission
- thorough peer review by experienced researchers in your field
- rapid publication on acceptance
- support for research data, including large and complex data types
- gold Open Access which fosters wider collaboration and increased citations
- maximum visibility for your research: over 100M website views per year

At BMC, research is always in progress.

Learn more biomedcentral.com/submissions

BMC
